# The effect of eggs on early child growth in rural Malawi: the Mazira Project randomized controlled trial

**DOI:** 10.1093/ajcn/nqz163

**Published:** 2019-08-06

**Authors:** Christine P Stewart, Bess Caswell, Lora Iannotti, Chessa Lutter, Charles D Arnold, Raphael Chipatala, Elizabeth L Prado, Kenneth Maleta

**Affiliations:** 1 Department of Nutrition, University of California, Davis, CA, USA; 2 School of Public Health and Family Medicine, College of Medicine, University of Malawi, Blantyre, Malawi; 3 Brown School, Institute for Public Health, Washington University in St. Louis, St. Louis, MO, USA; 4 RTI International, Washington DC, School of Public Health, University of Maryland, College Park, MD, USA

**Keywords:** eggs, child growth, Malawi, stunting, complementary feeding

## Abstract

**Background:**

Stunted growth is a significant public health problem in many low-income countries.

**Objective:**

The aim of this study was to evaluate the impact of 1 egg per day on child growth in rural Malawi.

**Design:**

We conducted an individually randomized controlled trial in which 660 children aged 6–9 mo were equally allocated into an intervention (1 egg/d) or control group. Eggs were provided during twice-weekly home visits for 6 mo. Control households were visited at the same frequency. Assessors blinded to intervention group measured length, weight, head circumference, and midupper arm circumference at baseline and the 6-mo follow-up visit. To assess adherence, multipass 24-h dietary recalls were administered at baseline, 3-mo, and 6-mo visits.

**Results:**

Between February and July 2018, 660 children were randomly assigned into the intervention (*n* = 331) and control (*n* = 329) groups. Losses to follow-up totaled 10%. In the intervention group, egg consumption increased from 3.9% at baseline to 84.5% and 70.3% at the 3-mo and 6-mo visits, whereas in the control group, it remained below 8% at all study visits. The baseline prevalence of stunting was 14%, underweight was 8%, and wasting was 1% and did not differ by group. There was no intervention effect on length-for-age, weight-for-age, or weight-for-length *z* scores. There was a significantly higher head circumference for age *z* score of 0.18 (95% CI: 0.01, 0.34) in the egg group compared with the control group. There was a significant interaction with maternal education (*P* = 0.024), with an effect on length-for-age *z* score only among children whose mothers had higher education.

**Conclusions:**

The provision of 1 egg per day to children in rural Malawi had no overall effect on linear growth. A background diet rich in animal source foods and low prevalence of stunting at baseline may have limited the potential impact. This trial was registered at clinicaltrials.gov as NCT03385252.

## Introduction

Poor growth in childhood is associated with an increased risk of infection, poor cognitive development, and lower economic opportunities in later life (1). Child stunting, as indicated by a low length-for-age *z* score (LAZ<-2), has been included as a Sustainable Development Goal target (2). Although there has been much effort focused on stunting prevention, the current rate of decline is insufficient to meet the target (3).

While all of the causal factors associated with growth faltering are not well understood, it is apparent that it begins early in life, starting *in utero* and continuing through at least the first 2 y of age. Diet quality appears to play a role, especially during the complementary feeding period (4). Poor dietary diversity is reflective of a lower nutrient density (5) and is associated with a greater risk of stunting (6). In particular, the consumption of animal source foods, such as eggs, dairy, meat, or fish, is associated with higher LAZ (7). Between the ages of 6 and 12 mo, when infants are first offered complementary foods, dietary quality and diversity are often extremely poor, sometimes reliant on just 1 staple food (8–10). Interventions focused on improving complementary feeding, however, have had only a modest impact on child growth (11), although few studies have directly tested the effect of animal source foods (12).

A study conducted in Ecuador among a population of children with a high prevalence of stunting found that providing 1 egg per day for 6 mo to young children during the early complementary feeding period was associated with a significant increase in LAZ of 0.63 (95% CI: 0.38, 0.88) and a 47% reduction in the prevalence of stunting (13). These results suggested that eggs might offer an opportunity to substantially improve early childhood growth. The study was small, however, with a total sample size of 163 children. There was some baseline imbalance in LAZ between groups, and it was unknown if the results would be broadly generalizable to other populations at risk of stunting. Therefore, it is important to replicate the study design in a different context. Thus, the design and overall objective for the present study in rural Malawi is highly comparable to the original trial in Ecuador: to evaluate the effect of daily consumption of eggs over a 6-mo period on child growth among children aged 6–9 mo at baseline. We hypothesized that children in the intervention group receiving 1 egg per day would have higher mean LAZ, weight-for-age *z* score (WAZ), weight-for-length *z* score (WLZ), and head circumference-for-age *z* score (HCAZ) compared with children in the control group.

## Subjects and Methods

### Study design and participants

The study was an individually randomized controlled trial (registered at clinicaltrials.gov as NCT03385252) carried out within the rural catchment areas of the Lungwena Health Center and St. Martins Hospital of Malindi, in Mangochi District, Malawi (**Supplementary Figure 1**). Study staff obtained a household listing of children from local health service assistants prior to the start of the trial. Children aged 6–9 mo residing in the catchment area were eligible for participation in the study. Recruitment occurred during community meetings and home visits to households with age-eligible children, and potentially eligible children were invited to a central location for screening and enrollment. The following criteria were used to screen prospective participants: infant age 6.0–9.9 mo, singleton child, midupper arm circumference (MUAC) >12.5 cm and no evidence of bipedal edema, hemoglobin >5 g/dL, no acute illness warranting hospital referral, no history of egg allergy and no reaction to egg during a test feeding, no history of anaphylaxis or severe allergic reaction to any substance, no congenital defects or chronic morbidity associated with growth or developmental impairments or that may affect feeding, and no plans to leave the study area in the next 6 mo. Enrollment occurred between February and July 2018, with follow-up visits occurring during the subsequent 6 mo.

A study staff member read a description of the study to all caregivers, typically mothers, of the eligible children during a group information session. After this session, caregivers met individually with a data collector in private and had the opportunity to review the consent statement and ask questions. If the mother agreed to enroll her child, she was asked to sign the consent form. All protocols were reviewed and approved by the institutional review boards at the University of California, Davis and the College of Medicine in Malawi.

### Randomization

Randomization occurred at the end of the baseline enrollment visit using a 1:1 allocation ratio in blocks of 10. The random sequence was generated by a researcher independent of the field team. Allocation codes were concealed in sealed, opaque envelopes. A study staff person invited mothers to select and open 1 envelope to reveal the child's allocation code. Randomization procedures were monitored by a community member independent of the study team who was nominated by local community leaders.

### Intervention

The intervention consisted of 1 egg per day for the study child for a period of 6 mo. The intervention was not blinded to participants or staff who conducted the household visits due to the nature of the product. The eggs were delivered twice-weekly by a study staff person during home visits. Each participating household was provided with a storage basket for the eggs, information about hygiene and handwashing during food preparation, recipes and suggestions for how to prepare eggs, and instructions not to share the child's eggs with other family members. Formative research in the study communities revealed that intrahousehold sharing was highly likely, particularly with siblings, and so the family was provided with an additional batch of 7 eggs per week that could be shared with other family members. Structured observations of egg intake were initially planned for home visits, but this was not feasible because most families provided the eggs to the child early in the morning prior to the arrival of the study personnel. During the second household visit each week, the staff person administered a 7-d morbidity history and brief FFQ focused on animal source foods. During the first 2 wk of the trial, a study staff person visited intervention households 4 d each week to provide additional messaging, coaching, and support for feeding the child eggs throughout the study.

The control group households were also visited twice per week and received messages about hygiene and handwashing during food preparation, but they did not receive eggs or any other foods during the study period. During the course of the trial, control households received participation incentives, including a bucket, laundry tub, and lidded plastic bin. At the end of the trial, they received a mixed basket of food items, including eggs. The total value of the incentives and food package matched the value of the eggs provided to the intervention households. During the first home visit each week, the staff person asked the caregiver to recall the child's most recent meal. Morbidity histories and FFQs were administered on the second visit.

### Outcome measures

At the baseline visit to the study clinic site, staff conducted a survey asking about child characteristics, conducted a multipass quantitative 24-h dietary recall, administered a set of developmental assessments, and performed anthropometric measurements. Dates of birth were recorded from the child's health passport issued by the local hospital or clinic (95.6% of children) or parental recall. A 5-mL blood sample was also collected. At the time of blood collection, staff measured hemoglobin concentrations (Hemocue 201, HemoCue Inc., Angelholm, Sweden) and tested for malaria using a rapid diagnostic test (SD Bioline Malaria Ag P.f/Pan, Abbott Diagnostics, Lake Forest, IL). Children with severe anemia or a positive malaria test were referred for treatment. A home visit was conducted during the following week to collect household socioeconomic and demographic information, as well as household food insecurity status (14). The Home Observation for Measurement of the Environment (HOME) inventory (15) was administered and the household Global Positioning System location was determined for future follow-ups. At the end of 6 mo, children were invited back to the study clinic, and the 24-h recall, developmental assessments, anthropometric measurements, and blood sample collection were repeated. Randomization occurred after the baseline assessments had been completed and the assessors who conducted the follow-up assessments were blinded to intervention group. This article focuses on the primary growth outcome measurements. Other study outcomes will be reported elsewhere.

Trained and standardized anthropometrists working in pairs conducted all measurements in triplicate. Child recumbent length was measured to the nearest millimeter using a Holtain length board (Holtain Ltd.); weight was measured in the caregiver's arms using the tare function of the Seca 874 digital scale (Seca); MUAC and head circumference were measured using an insertion tape. The measuring tapes were replaced between the baseline and 6-mo assessments. Tapes were from Health Books International at baseline and Seca (model 212) at the follow-up visit. Maternal weight and standing height were also measured at baseline. Child LAZ, WAZ, WLZ, and HCAZ were calculated using the WHO Growth Standards (16). We screened for biologically implausible outliers using the following cut-offs: LAZ<–6 or >6, WAZ<–6 or >5, WLZ<–5 or >5, and HCAZ<–5 or >5. This resulted in dropping 1 baseline head circumference value and its corresponding *z* score. Dichotomous variables were created for stunting (LAZ<–2), underweight (WAZ<–2), wasting (WLZ<–2), and low head circumference (HCAZ<–2).

Dietary intake was monitored using a tablet-based multipass 24-h dietary recall conducted in both groups at baseline, a 3-mo home visit, and the 6-mo clinic visit. In a subsample of 100 per group, replicate recalls were conducted on up to 2 additional days for each visit time point.

### Statistical analysis

The sample size for the trial was based on a hypothesized effect of a 0.25 difference in LAZ between the intervention and control group over the 6-mo period, assuming a 2-sided α = 0.05, power of 80%, and 80% adherence to the intervention. This effect size was considered comparable to other trials of complementary feeding practices and about half that which was observed during the similar trial in Ecuador (13).

A detailed statistical analysis plan was developed prior to the end of the study and was posted publicly (https://osf.io/xzufd/). Stata (version 14.1; StataCorp LLC) was used for data cleaning and management (17). Statistical analyses were performed using R (version 3.5.0; R Foundation for Statistical Computing) (18). The primary analysis relied on a complete-case, intention-to-treat comparison between the 2 groups. The primary outcome parameters of interest were the mean difference in LAZ and prevalence ratio (19) of stunting comparing the intervention group with the control group. All other anthropometric measures were considered secondary. Continuous outcome measures were analyzed using linear regression models, controlling for the baseline measure of the outcome variable. Dichotomous outcome variables were analyzed as prevalence ratios and prevalence differences, also adjusting for baseline values. The ratios were calculated using a binomial distribution with a log link or modified Poisson regression when a model failed to converge, while linear probability models were used for the estimation of prevalence differences (19, 20). *P* values <0.05 were considered statistically significant. Data analysis code was developed while blinded to intervention groups by using an independently created set of randomization codes that did not match actual group assignment. Data were unblinded after the last participant visit and after all of the primary data analysis code had been written.

For each outcome measure, we conducted a secondary adjusted analysis considering a prespecified list of covariates, including child age at assessment, sex, and birth order; maternal age, height, education, literacy, marital status, tribe, occupation, and religion; household asset index (21); number of children <5 y old; household food insecurity access score (14); animal ownership; distance to water source; field staff member who completed the measurement; month of outcome assessment; and closest health center. We prescreened covariates in bivariate models to assess whether they were associated with the outcome prior to including them in the adjusted models. Covariates with a *P* value <0.1 were included in the analysis. Of the variables considered, distance to water source and goat ownership were not associated with any outcome measures and therefore were not included in any analysis. We considered the potential for effect modification on the additive scale by testing an interaction term between the intervention group and child sex, birth order, maternal age, maternal education, baseline household food insecurity, baseline socioeconomic status, and baseline LAZ. Interactions with a *P* value <0.1 were considered statistically significant. In post-hoc exploratory analysis, we examined the relation between the interaction term and other baseline characteristics as well as indicators of adherence to the intervention.

We examined missing data in the following ways. We examined whether losses to follow-up were balanced across groups. We compared baseline characteristics of children who were lost to follow-up or who had missing data on the primary outcome variable with those who had complete data. Because the rate of missing data was low (∼10%) and not differential by study arm, we did not impute missing values.

## Results

**FIGURE 1:**
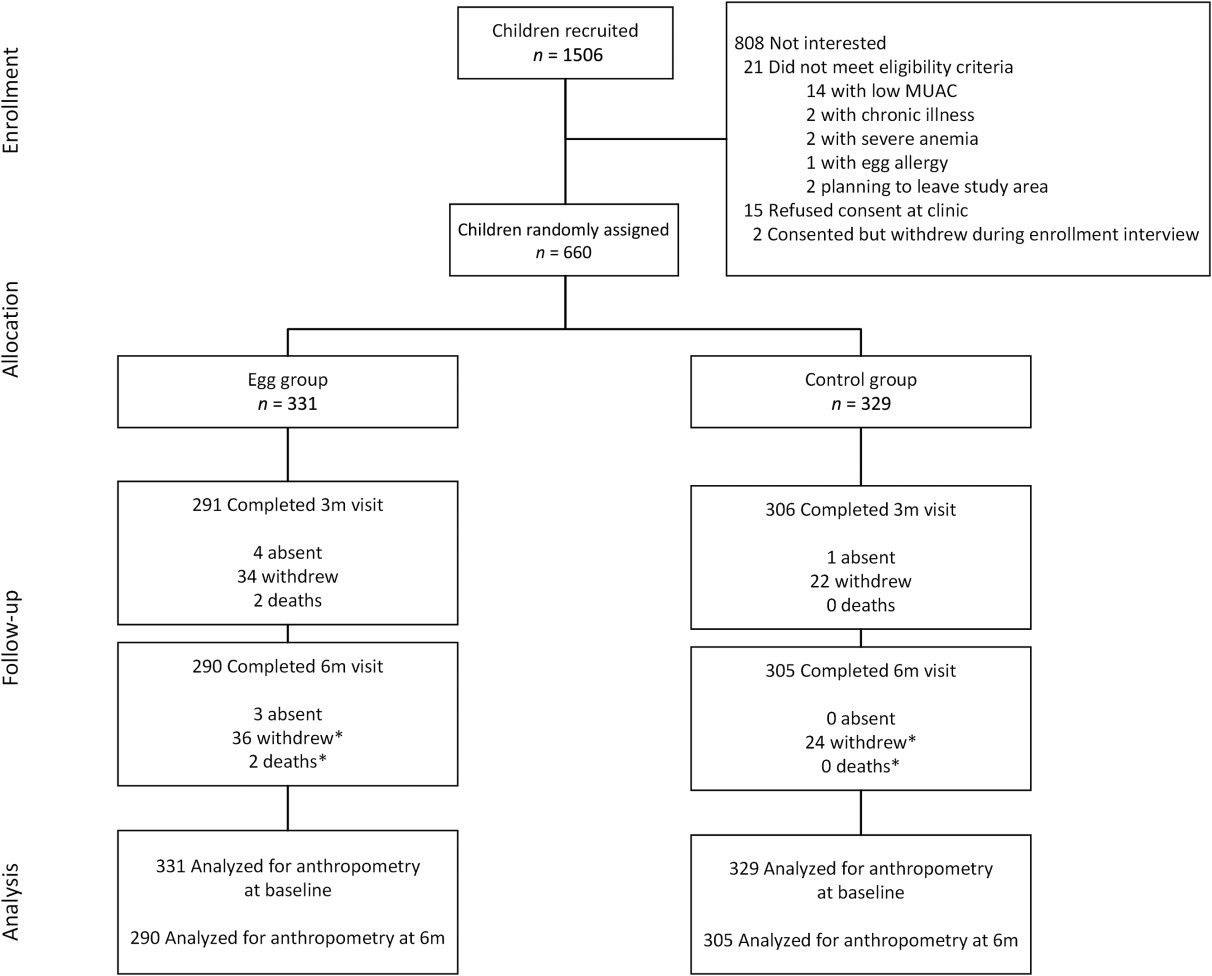
Participant flow diagram. *Numbers are cumulative. MUAC, midupper arm circumference.

A total of 1506 children were approached for recruitment into the study. Of these, 660 met the eligibility criteria, had a caregiver consent to their participation (99% were mothers), and were randomly allocated into the 2 groups (**Figure 1**). Over the course of the study, the overall loss to follow-up rate in the 2 groups was 10%: a total of 60 children withdrew, 3 were absent, and 2 died. The losses were slightly higher in the intervention group (12%) than the control group (7%). The baseline characteristics of those included in the analysis and those with missing data differed by education, literacy, health center location, and housing characteristics (**Supplementary Table 1**). Those who dropped out tended to have lower socioeconomic status compared with those who remained in the study. All other characteristics were generally similar.

Children were a mean ± SD age of 7.4 ± 1.1 mo, and the majority had at least 1 older sibling (**Table 1**). Only 46% of mothers were able to read, and the majority had not completed primary schooling. The proportion with no formal education was slightly higher in the control group (21.9%) compared with the intervention group (14.2%). The prevalence of child malaria was ∼13%, and anemia was common (∼60%). Breastfeeding was nearly universal. Reported household food insecurity was common, with ∼70% reporting severe food insecurity at baseline. Slightly more than half of children were recruited from the Lungwena Health Center catchment area in the north, and the remainder were recruited from the St. Martins Hospital of Malindi catchment area in the south (Supplementary Figure 1). The baseline characteristics were well balanced between the 2 groups.

**TABLE 1:** Enrollment characteristics by intervention group

	Control		Egg	
Characteristic	*n*	% or Mean ± SD	*n*	% or Mean ± SD
Maternal				
Maternal age (y)	325	26.1 ± 6.8	329	25.9 ± 6.7
Maternal BMI (kg/m^2^)	329	21.8 ± 2.8	331	21.8 ± 3.2
Maternal education (% completed primary or greater)	329	16.4	331	23.6
Maternal marital status				
Monogamous	329	59.9	331	55.6
Polygamous		21.3		18.7
Unmarried		18.8		25.7
Maternal tribe				
Chewa or other	321	15.3	322	13.7
Yao		84.7		86.3
Maternal occupation				
Farming or fishing	320	42.2	322	44.4
Housewife		35.9		31.1
Service		21.9		24.5
Child				
Child age, mo	329	7.3 ± 1.2	331	7.4 ± 1.2
Sex (% female)	329	48.3	331	48.3
Birth order (% first born)	329	25.2	330	30.0
Malaria	296	12.5	299	12.7
Anemia	290	61.4	292	59.9
Breastfeeding	329	100.0	330	99.7
Household				
Nearest health center				
Lungwena	329	52.6	331	53.8
Malindi		47.4		46.2
Religion (% Muslim)	321	88.2	322	87.9
Paternal occupation				
Farming or fishing	272	47.1	244	50.0
Service		52.9		50.0
HOME inventory score^1^	321	24.0 ± 3.5	322	24.4 ± 3.5
Number of children under 5 y	319	1.7 ± 0.8	319	1.7 ± 0.8
Number of household members	320	6.0 ± 2.7	321	5.8 ± 2.6
Moderate or severe food insecurity^2^	329	81.2	331	74.6
Own latrine	321	96.6	322	96.3
Distance to water source (% <10 min)	321	54.5	322	56.8
Poor floor quality^3^	321	76.0	322	77.0
Poor roof quality^3^	321	62.3	322	59.6
Poor wall quality^3^	321	44.2	322	43.5
Any cows owned	321	2.8	322	3.1
Any goats owned	329	20.4	329	17.6
Any chickens owned	329	35.6	330	29.1

1 HOME, Home Observation for Measurement of the Environment (15).

2 Food insecurity assessed using the Household Food Insecurity Access Scale (14).

3 Poor quality defined as straw, grass, mud, or unburnt brick.

At baseline, ∼4% of children were reported to have consumed eggs on the previous day (**Table 2**). This increased to 85% at the 3-mo visit and 71% at the 6-mo visit in the egg intervention group. In the control group, the proportion remained low at 6–7% throughout. The consumption of dairy foods was low at baseline but increased to about 19% in both groups by the 6-mo visit. Meat consumption remained below 10% at all time points. In contrast, fish consumption was between 22% and 32% at baseline, increasing to ∼60–65% at the 3-mo and 6-mo visits in both groups.

**TABLE 2:** Animal source food consumption by intervention group^1^

	Control		Egg	
Characteristic	*n*	%	*n*	%
Consumed eggs				
Enrollment	329	4.0	330	4.2
3 mo	306	6.5	291	84.9
6 mo	305	7.2	290	71.0
Consumed fish				
Enrollment	329	22.5	330	31.8
3 mo	306	64.7	291	59.8
6 mo	305	60.7	290	65.5
Consumed dairy				
Enrollment	329	9.4	330	7.6
3 mo	306	12.4	291	10.0
6 mo	305	19.7	290	18.6
Consumed meat				
Enrollment	329	1.5	330	2.1
3 mo	306	6.5	291	6.5
6 mo	305	8.9	290	9.7

1 Any consumption reported in a 24-h recall period.

Children had a mean ± SD length of 66.8 ±2.9 cm, weight of 7.7 ± 1.0 kg, and head circumference of 42.2 ± 1.7 cm at baseline that increased to 73.7 ± 2.9 cm, 9.0 ± 1.1 kg, and 45.6 ± 1.6 cm at the 6-mo follow-up. The baseline mean ± SD LAZ did not differ between groups: –0.86 ± 0.99 in the control group and –0.91 ± 1.05 in the egg group (**Table 3**). Baseline values of WAZ, WLZ, and HCAZ were also comparable between groups. At the end of the 6-mo intervention, there was no effect on LAZ. There was a mean difference in LAZ of 0.07 (95% CI: –0.01, 0.15) between groups when adjusting for baseline values, and this did not change after adjusting for additional covariates. There was also no effect on the prevalence of stunting (prevalence ratio: 0.98; 95% CI: 0.80, 1.19). Similarly, there were no effects on WAZ or WLZ or on the prevalence of underweight or wasting. There was a significant effect on HCAZ. The mean difference between groups was 0.18 (95% CI: 0.01, 0.34) after controlling for baseline values. Because the measuring tapes had been replaced between baseline and follow-up, we also analyzed the data without adjusting for baseline. The unadjusted mean difference was 0.23 (95% CI: 0.05, 0.41) units higher in the egg group; adjusting for additional covariates did not substantively change this estimate (0.22; 95% CI: 0.06, 0.39).

**TABLE 3:** Mean differences and prevalence ratios comparing the egg intervention with the control group^1^

	Baseline		6 mo follow-up		Minimally adjusted^2^ comparison (95% CI)	Fully adjusted^3^ comparison (95% CI)
Characteristic	Control (*n* = 329)	Egg (*n* = 331)	Control (*n* = 305)	Egg (*n* = 290)		
LAZ	–0.86 (0.99)	–0.91 (1.05)	–1.10 (1.00)	–1.08 (1.08)	0.07 (–0.01, 0.15)	0.07 (–0.01, 0.14)
Stunted, %	14.0	13.3	19.0	19.3	0.98 (0.80, 1.19)	1.04 (0.79, 1.36)
WAZ	–0.57 (1.04)	–0.50 (1.12)	–0.74 (1.00)	–0.61 (1.11)	0.07 (–0.02, 0.16)	0.08 (–0.01, 0.16)
Underweight, %	8.5	7.3	11.8	11.7	1.10 (0.86, 1.42)	1.04 (0.73, 1.47)
WLZ	–0.02 (1.00)	0.12 (1.07)	–0.30 (0.97)	–0.13 (1.04)	0.06 (–0.04, 0.17)	0.06 (–0.04, 0.16)
Wasted, %	1.2	0.9	2.0	3.4	1.90 (0.72, 5.05)	2.31 (0.95, 5.62)
HCAZ	–1.13 (1.11)	–1.01 (1.12)	–0.33 (1.09)	–0.10 (1.18)	0.18 (0.01, 0.34)	0.12 (0.03, 0.21)
Small head size, %	22.2	18.2	7.9	5.5	0.74 (0.40, 1.36)	0.84 (0.49, 1.42)

1 Values are mean (SD) with difference in means for comparison or prevalence and prevalence ratio for comparison. HCAZ, head circumference-for-age *z* score; LAZ, length-for-age *z* score; WAZ, weight-for-age *z* score; WLZ, weight-for-length *z* score.

2 Adjusted for baseline value.

3 Adjusted for baseline value and potentially adjusted for child age at measurement, sex, birth order, maternal age, height, education, literacy, marital status, tribe, occupation, religion, number of children younger than 5 y in the household, food security, housing and asset index, animal ownership, distance to water source, field staff who performed measurements, month of measurement, and closest health center.

**FIGURE 2:**
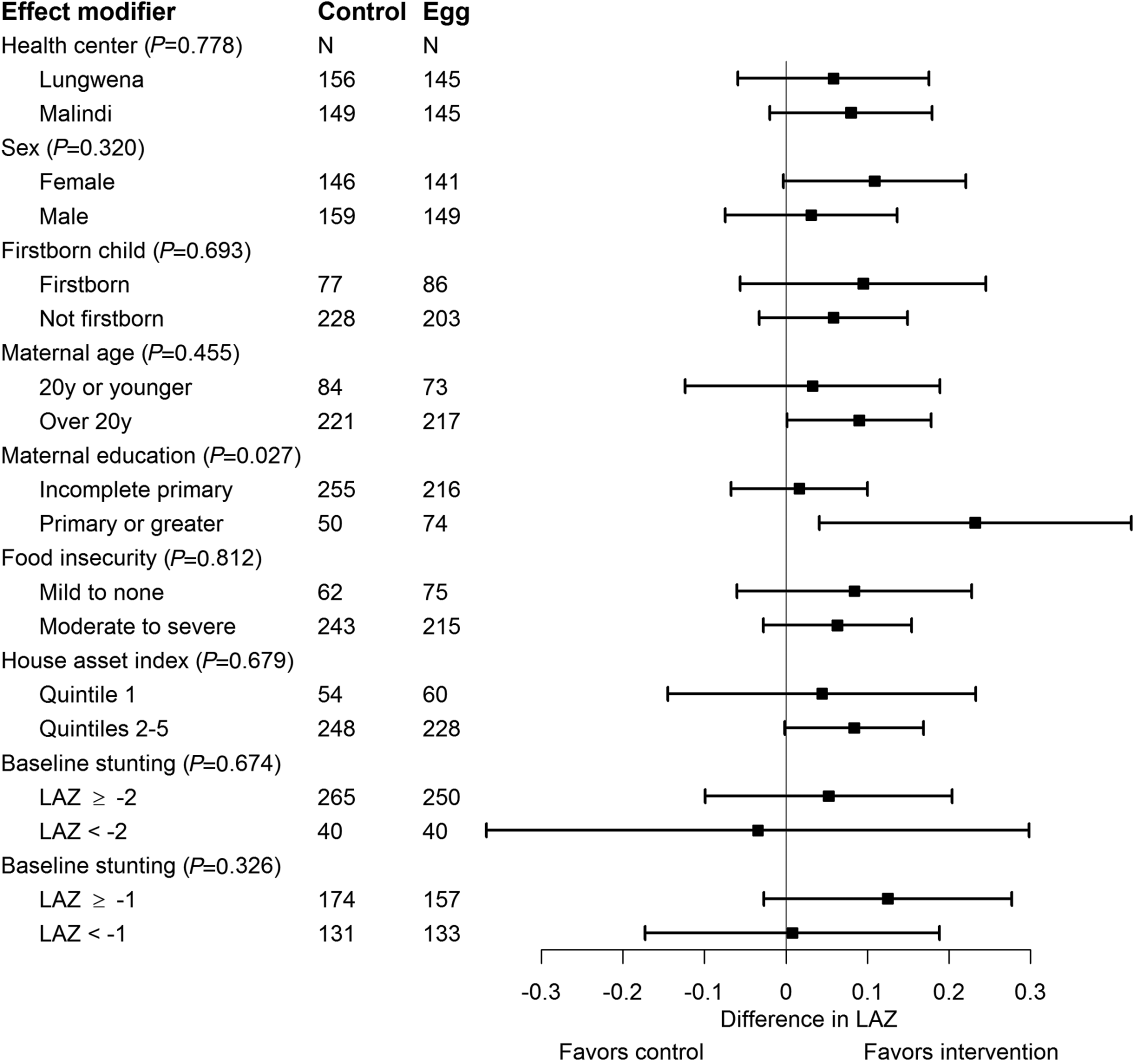
The effect of the egg intervention on length-for-age *z* score stratified on prespecified effect modifiers of interest. LAZ, length-for-age *z* score.

We found only 1 significant effect modification between maternal education and intervention group (*P* = 0.027, **Figure 2**). There was a significant intervention effect among children whose mothers had higher levels of education, an increase of 0.23 (95% CI: 0.04, 0.42) in LAZ in the egg group relative to the controls. There was no effect of the intervention among children whose mothers had lower levels of education, however. Adherence rates were comparable between those with higher education and lower education (**Supplementary Table 2**). However, many other characteristics differed by maternal educational level (**Supplementary Table 3**). Mothers with higher levels of education had slightly higher BMI, were more likely to be of the Chewa tribe, and had slightly higher HOME inventory scores. They were less likely to be fishermen, to be food insecure, and to have poor-quality homes. Their children were more likely to be first born, less likely to have malaria, and more likely to have consumed dairy or eggs on the previous day at baseline. At the 6-mo visit, they were less likely to have consumed fish. Throughout the trial, their children also had a slightly lower percentage of days with symptoms of fever, diarrhea, and bloody stool.

## Discussion

In this randomized controlled trial in rural Malawi with low attrition and high rates of adherence, we found that the provision of 1 egg per day for 6 mo offered no overall benefit on length gain or risk of stunting. Nevertheless, there was a significant effect on HCAZ and evidence of effect modification by maternal education, with effects on LAZ concentrated in children whose mothers had higher levels of education. It is notable that this study was conducted in close proximity to Lake Malawi, an environment where fish consumption is common. Nearly two-thirds of children were reported to consume fish on the previous day at the 3-mo and 6-mo visits. In a cohort study in a separate region of Malawi, the consumption of animal source proteins, primarily from fish, was significantly associated with HAZ (22). The results from this trial provide an addition to the literature on the effects of animal source foods on early child growth (12) and may suggest that eggs offer limited added benefit on child growth when other high-quality animal source foods are readily accessible.

A key question is why the intervention offered no growth benefit in Malawi, whereas a similar intervention resulted in significant increases in LAZ in the highlands of Ecuador (13). A number of contextual differences between the 2 locations might offer some clues. First, the mean LAZ at baseline was lower in the Ecuador trial (LAZ = –1.9) compared with the present study (LAZ = –0.9) (**Supplementary Figure 2**). The Ecuador Lulun Project trial was conducted in a mixed indigenous community, where there are substantial disparities from the national averages in terms of health and socioeconomic conditions. The 2013 National Health and Nutrition Survey reported that the prevalence of stunting among indigenous children was 42% (23), comparable to that reported in the Lulun Project but higher than the national average of 25%. It is notable that the present study cohort in Malawi also had a higher mean LAZ at 6–9 mo than a previous study cohort (LAZ = –1.3 at 6 mo in the control group) that was measured in the same study communities just 7 y ago (24). Second, consumption of fish was very common in this site in Malawi. In both groups, ∼60% of children consumed fish at the 3-mo or 6-mo follow-up surveys, whereas in Ecuador, animal source food consumption aside from eggs was infrequent. In the Ecuador sample, ∼20% consumed fish on the previous day at the 6-mo follow-up (L Iannotti, personal communication, Washington University in St. Louis, 2019). Both of these 2 issues may have limited the potential for children to benefit in the Malawi site. There are also differences in staple food consumption between the communities: in Ecuador, potatoes and rice were most common, whereas in Malawi, maize was predominant. Other factors, such as the burden and types of infections, might also differ between these contexts. In Ecuador, 73% reported having treated water and 89% had flush toilets (13). In contrast, in Malawi, only 20% of households reported treating their water and 0 had flush toilets, increasing risks for enteric infection. In Ecuador, the heavier burden of infectious disease arose from respiratory infections. Maternal educational levels were also higher in Ecuador, where primary school completion was nearly universal. In Malawi, only 20% had completed primary schooling.

Subgroup analyses suggested that there was some benefit of the intervention on LAZ among children of more highly educated mothers. This effect did not appear to be attributable to greater adherence in the more highly educated families. There were many differences in characteristics between households with higher and lower education, however. In particular, housing quality was generally better overall, and there was about half the prevalence of malaria at baseline. Children with more highly educated mothers were less likely to have reported symptoms of illness, suggesting they were less likely to have other infections as well, a finding that has been seen in other contexts (25, 26). The overall magnitude of the difference in morbidity symptoms was small, however. Nevertheless, this may enable these children to be more responsive to the intervention, when their growth is not constrained by infection or inflammation. The children with more highly educated mothers were also less likely to consume fish at the 6-mo visit but more likely to consume dairy products at every visit. Future analyses will examine morbidity symptoms, markers of inflammation, and dietary practices, including the potential for displacement of other complementary foods, in this study cohort.

The study suffered from a few weaknesses. Egg consumption patterns were reported, not directly observed. Intervention households may have chosen to sell or share the eggs provided by the study, but direct spillovers from the intervention to control households appeared unlikely. Second, we noted problems with the head circumference tapes used in the baseline assessments, which appeared to wear out quickly. They were replaced with a more robust tape between the baseline and follow-up surveys, which likely led to systematic measurement error between survey rounds. Both study groups were measured using the same tapes, and anthropometrists were blinded to treatment assignment. Therefore, we believe that the between-group differences in head circumference are valid estimates, but the change over time from baseline to endline likely is not.

The strengths of this study include its large sample size and high rate of follow-up, which offered sufficient power to detect small effects. The fidelity of the intervention delivery was strong—there was a large-magnitude difference in egg consumption frequency between the intervention and control groups. There was also a high degree of support and encouragement provided to study households, including twice-weekly household visits and intensive coaching provided during the first 2 study weeks. Because of the potential for intrahousehold sharing, an additional batch of 7 eggs was provided to each household weekly, on top of the weekly ration for the index child. The anthropometric measurements were conducted by highly trained and standardized field workers who were blinded to the intervention group throughout the study. The statistical analysis plan was prespecified and publicly posted, and all analyses were conducted on blinded data sets.

In summary, we found that the provision of 1 egg per day for 6 mo to young children had no overall effect on linear growth in this rural Malawian context. The impact of egg consumption may differ in populations with a higher prevalence of stunting, in which the intake of animal source foods is uncommon, where household water and sanitation conditions are improved, or in populations with higher levels of education. To fully understand the potential nutritional impacts of eggs in key target groups, additional rigorous well-designed studies are needed that can evaluate the efficacy in populations that might have a greater potential to benefit and respond to the intervention.

## Supplementary Material

nqz163_Supplemental_File

## Notes

This study was funded by the Bill and Melinda Gates Foundation (OPP1169033).

Supplemental Tables 1–3 and Supplemental Figures 1 and 2 are available from the “Supplementary data” link in the online posting of the article and from the same link in the online table of contents at https://academic.oup.com/ajcn/.

Abbreviations used: HCAZ, head circumference-for-age *z* score; HOME, Home Observation for Measurement of the Environment; LAZ, length-for-age *z* score; MUAC, midupper arm circumference; WAZ, weight-for-age *z* score; WLZ, weight-for-length *z* score.
