# Supplementary material for: The effect of eggs on early child growth in rural Malawi: the Mazira Project randomized controlled trial
Source: Am J Clin Nutr. 2019 Aug 6;110(4):1026–33. doi: 10.1093/ajcn/nqz163 (PMC6766435; doi:10.1093/ajcn/nqz163)
Supplement: nqz163_Supplemental_File [file nqz163_supplemental_file.pdf]

The effect of eggs on early child growth in rural Malawi: the Mazira Project randomized controlled trial

Christine P. Stewart, Bess Caswell, Lora Iannotti, Chessa Lutter, Charles D. Arnold, Raphael Chipatala, Elizabeth L. Prado, Kenneth Maleta

Supplementary Material

Table of Contents:

Supplementary Figure 1: Map of study site

Supplementary Table 1: Enrollment characteristics by inclusion in analysis

Supplementary Table 2: Egg consumption by education category and intervention group

Supplementary Table 3: Characteristics by maternal education category

Supplementary Figure 2: Comparison of mean (95% CI) length-for-age z-score (LAZ) between the Malawi Mazira Project, Ecuador Lulun Project, and Malawi iLiNS Project cohorts.

Supplementary Figure 1: Map of study site

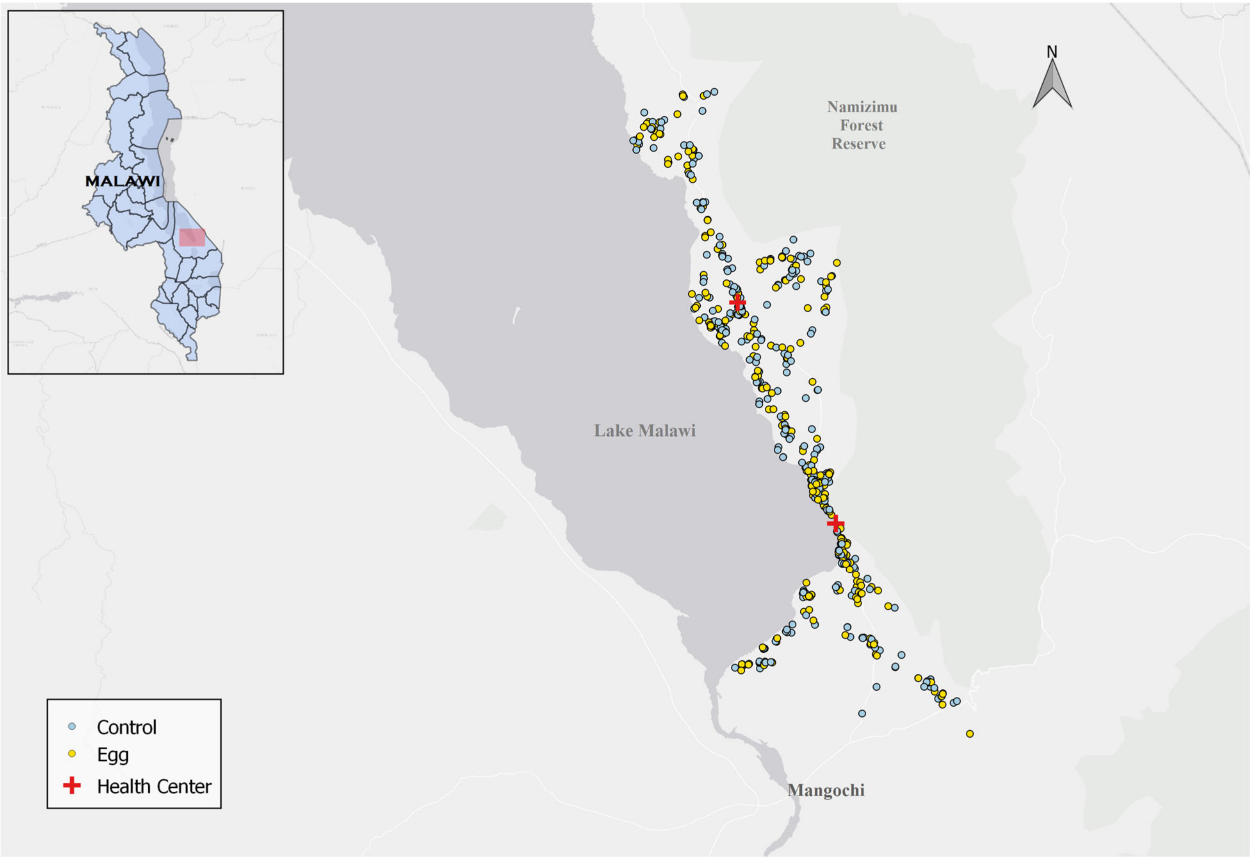

Supplementary Table 1: Enrollment characteristics by inclusion in analysis[1]

Characteristics		Has endline		No endline		
		N	% or Mean (SD)	N	% or Mean (SD)	p-value
**Maternal**						
Maternal age (y)		595	26.1 (6.7)	61	24.6 (7.3)	0.079
Maternal BMI (kg/m^2)		595	21.8 (3.1)	65	21.6 (2.7)	0.577
Maternal education	Completed primary or greater	595	20.8	67	11.9	0.068
Mother can read		592	47.1	51	31.4	0.028
Maternal marital status	Monogamous	595	57.1	67	64.2	0.284
	Polygamous		19.8		20.9	
	Unmarried		23.0		14.9	
Maternal tribe	Chewa or other	592	15.0	51	7.8	0.132
	Yao		85.0		92.2	
Maternal occupation	Farming or fishing	591	42.3	51	54.9	0.125
	Housewife		33.7		31.4	
	Service		24.0		13.7	
**Child**						
Child age (m)		595	7.4 (1.2)	67	7.6 (1.1)	0.166
Female		595	48.2	65	49.2	0.879
Firstborn		594	27.4	65	29.2	0.761
Malaria		538	12.6	59	11.9	0.864
Anemia		528	61.0	56	58.9	0.765
Breastfeeding		594	99.8	65	100.0	0.648
**Household**						
Health center	Lungwena	595	50.6	67	77.6	0.000
	Malindi		49.4		22.4	
Muslim		592	87.7	51	92.2	0.318
Paternal occupation	Farming or fishing	472	48.9	44	43.2	0.464
	Service		51.1		56.8	
HOME inventory score[2]		592	24.2 (3.5)	51	24.1 (3.2)	0.858
Number of children under 5y		587	1.7 (0.8)	51	1.8 (0.8)	0.763
Number of household members		591	5.9 (2.6)	50	5.9 (3.4)	0.925
Moderate or severe food insecurity[3]		595	77.0	65	86.2	0.076
Own latrine		592	96.6	51	94.1	0.393
Distance to water source	<10 min	592	55.7	51	54.9	0.908
Poor floor quality[4]		592	75.7	51	86.3	0.070
Poor roof quality[4]		592	60.0	51	72.5	0.071
Poor wall quality[4]		592	43.1	51	52.9	0.175
Any cows owned		592	2.7	51	5.9	0.250
Any goats owned		595	19.7	65	12.3	0.132
Any chickens owned		595	32.6	66	30.3	0.703

[1]% or Mean (SD)
[2]Home Observation for Measurement of the Environment (Caldwell & Bradley, 2003).
[3]Food insecurity assessed using the Household Food Insecurity Access Scale (Coates et al, 2007).
[4]Poor quality defined as straw, grass, mud or unburnt brick.

Supplementary Table 2: Egg consumption by education category and intervention group[1]

Timepoint and education	Control	Egg
**Baseline**		
Incomplete primary	2.4	2.8
Primary or greater	10.0	8.2
**Midline**		
Incomplete primary	5.5	83.6
Primary or greater	12.0	87.8
**Endline**		
Incomplete primary	6.3	71.8
Primary or greater	12.0	68.9

[1]% with any egg consumption by 24hr recall.

Supplementary Table 3: Characteristics by maternal education category[1]

Characteristics		Incomplete primary N	% or Mean (SD)	Primary or greater N	% or Mean (SD)	p-value
**Household**						
Health center	Lungwena	471	55.0	124	33.9	0.000
	Malindi		45.0		66.1	
Muslim		468	93.8	124	64.5	0.000
Paternal occupation	Farming or fishing	376	54.8	96	26.0	0.000
	Service		45.2		74.0	
HOME inventory score[2]		468	24.0 (3.5)	124	25.0 (3.4)	0.006
Number of children under 5y		463	1.8 (0.8)	124	1.6 (0.8)	0.011
Number of household members		467	5.9 (2.6)	124	5.8 (2.6)	0.632
Moderate or severe food insecurity[3]		471	81.1	124	61.3	0.000
Own latrine		468	95.7	124	100.0	0.002
Distance to water source	<10 min	468	55.3	124	57.3	0.702
Number of rooms in home		467	2.6 (1.2)	123	3.4 (1.1)	0.000
Poor floor quality[4]		468	82.9	124	48.4	0.000
Poor roof quality[4]		468	67.7	124	30.6	0.000
Poor wall quality[4]		468	50.2	124	16.1	0.000
Any cows owned		468	2.6	124	3.2	0.692
Any goats owned		471	20.0	124	18.5	0.724
Any chickens owned		471	31.6	124	36.3	0.328
**Maternal**						
Maternal age (y)		471	26.3 (6.8)	124	25.5 (6.1)	0.261
Maternal BMI (kg/m^2)		471	21.7 (2.9)	124	22.4 (3.6)	0.016
Mother can read		468	33.1	124	100.0	0.000
Maternal marital status	Monogamous	471	56.3	124	60.5	0.227
	Polygamous		21.2		14.5	
	Unmarried		22.5		25.0	
Maternal tribe	Chewa or other	468	8.5	124	39.5	0.000
	Yao		91.5		60.5	
Child's primary language	Chichewa	466	10.9	123	48.8	0.000
	Chiyao		89.1		51.2	
Maternal occupation	Farming or fishing	467	45.8	124	29.0	0.003
	Housewife		31.5		41.9	
	Service		22.7		29.0	
**Child**						
Child age (m)		471	7.4 (1.2)	124	7.2 (1.1)	0.252
Female		471	47.3	124	51.6	0.398
Breastfeeding		471	100.0	123	99.2	0.076
Firstborn		470	23.4	124	42.7	0.000
Malaria		427	14.1	111	7.2	0.041
Anemia		419	62.5	109	55.0	0.156
**Diet at baseline[5]**						
Eggs		471	2.5	123	8.9	0.003
Fish		471	26.1	123	26.0	0.982
Dairy		471	6.8	123	17.1	0.001
Meat		471	1.5	123	4.1	0.097
**Diet at midline[5]**						
Fish		468	62.8	124	61.3	0.755
Dairy		468	9.0	124	19.4	0.002
Meat		468	5.8	124	9.7	0.136
**Diet at endline[5]**						
Fish		471	65.2	124	54.8	0.035
Dairy		471	17.2	124	26.6	0.022
Meat		471	8.7	124	11.3	0.387
**Percent of time with illness[6]**						
Fever		470	10.7 (8.0)	123	9.1 (7.8)	0.038
Diarrhea		470	12.5 (9.4)	123	10.5 (9.0)	0.034
Bloody stool		470	0.5 (1.5)	123	0.2 (1.0)	0.031
Cough		470	17.7 (12.2)	123	15.4 (11.0)	0.059
Nasal discharge		470	21.2 (14.2)	123	19.1 (11.8)	0.140
Wheezing		470	3.3 (5.0)	123	3.0 (4.1)	0.607
Ear infection		470	3.1 (6.0)	123	2.1 (6.9)	0.085

[1]% or Mean (SD)
[2]Home Observation for Measurement of the Environment (Caldwell & Bradley, 2003).
[3]Food insecurity assessed using the Household Food Insecurity Access Scale (Coates et al, 2007).
[4]Poor quality defined as straw, grass, mud or unburnt brick.
[5]Any consumption, based on 24hr recall.
[6]Number of days with reported symptom of illness divided by total number of recall days. Assessed using morbidity recalls administered weekly throughout the trial.

Supplementary Figure 2: Comparison of mean (95% CI) length-for-age z-score (LAZ) between the Malawi Mazira Project, Ecuador Lulun Project, and Malawi iLiNS Project cohorts.

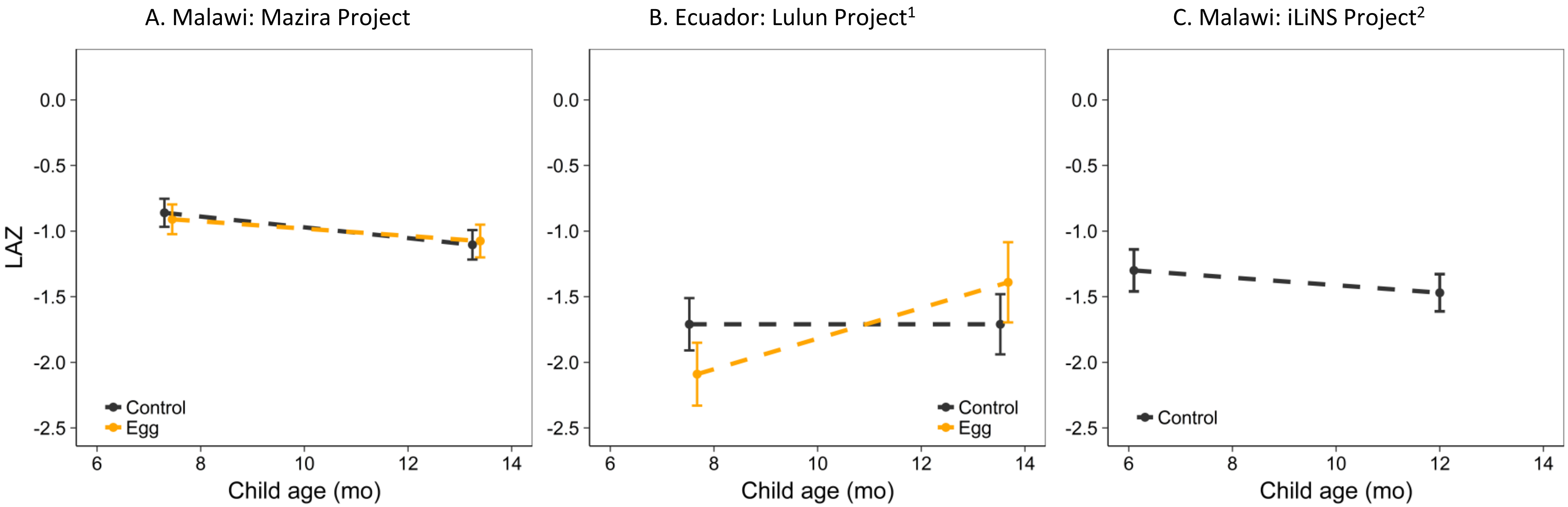

1. Data extracted from Iannotti et al. Eggs in Early Complementary Feeding and Child Growth: A Randomized Controlled Trial. Pediatrics 2017; 140(1). doi: 10.1542/peds.2016-3459.
2. Data extracted from Ashorn et al. Supplementation of maternal diets during pregnancy and for 6 months postpartum and infant diets thereafter with small-quantity lipid-based nutrient supplements does not promote child growth by 18 months of age in rural Malawi: a randomized controlled trial. J Nutr 2015;145(6):1345-53. doi: 10.3945/jn.114.207225.
